# Protocol for unbiased, consolidated variant calling from whole exome sequencing data

**DOI:** 10.1016/j.xpro.2022.101418

**Published:** 2022-05-30

**Authors:** Kleio-Maria Verrou, Georgios A. Pavlopoulos, Panagiotis Moulos

**Affiliations:** 1 Center of New Biotechnologies & Precision Medicine, Medical School, National and Kapodistrian University of Athens, Athens, Greece; 2 Institute for Fundamental Biomedical Research, Biomedical Sciences Research Center ‘Alexander Fleming’, Vari, Greece

**Keywords:** Bioinformatics, Genetics, Genomics

## Abstract

Whole Exome Sequencing (WES) is used for querying DNA variants using the protein coding parts of genomes (exomes). However, WES analysis can be challenging because of the complexity of the data. Here, we describe a consolidated protocol for unbiased WES analysis. The protocol uses three variant callers (HaplotypeCaller, FreeBayes, and DeepVariant), which have different underlying models. We provide detailed execution steps, as well as basic variant filtering, annotation, visualization, and consolidation aspects.

## Highlights

• Protocol to enable whole exome data analysis in an unbiased approach
• A protocol for unbiased analysis using 3 variant callers with different underlying models
• From raw data to filtered, consolidated, and annotated DNA variant calls

## Abstract (editor highlights)

Publisher’s note: Undertaking any experimental protocol requires adherence to local institutional guidelines for laboratory safety and ethics.

## Graphical abstract

**Figure:**
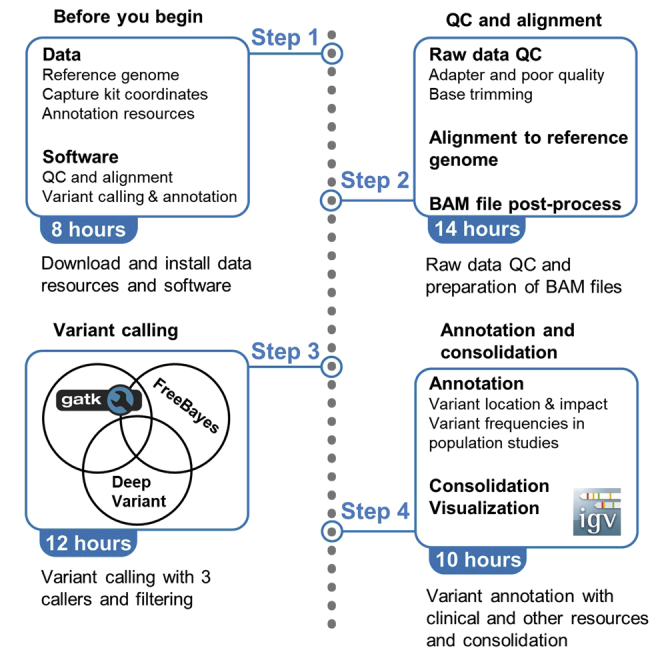

## Before you begin

The consolidated variant and annotation calling process presented in this protocol uses three variant callers, namely the HaplotypeCaller component of the Genome Analysis Toolkit 4.0 (DePristo et al., 2011), FreeBayes (Garrison and Marth, 2012) and DeepVariant (Poplin et al., 2018). It also provides instructions for general clinical variant annotation and addition of variant frequencies from major population studies, as well as steps to properly intersect and/or unify the (filtered and annotated) variant calls from each algorithm. In order to execute the protocol, apart from the basic software tools, additional resources are required, which can be categorized in reference file downloads (reference genome, genomic coordinates of the kit used for WES, reference annotation databases) and additional software tools for the pre- and post-processing of the input and output data respectively. The protocol is applied on a number of human datasets of common interest. Generally, we follow published and widely accepted best practices, with minor divergences. One example of divergence is that while the GATK community suggests using their own tools for preparing reference genome-aligned (BAM) files for variant calling, we use samtools which perform the pre-processing but are significantly faster and in harmony with the rest of the variant callers. The following steps apart from Data Collection can be performed once as they concern online data and software resources that can be stored locally. In addition, all steps assume a basic familiarity with the Unix/Linux command line, as all the commands are executed in the command line via a terminal.

### Resources download

**Timing: 6 h**

In this section, the required non-software resources (reference genome, annotation files) are retrieved. In the end of the section these resources should be in the proper places, ready for later usage.

1. Set the directory where the reference genome and genomic annotations will be placed for later and general use.

RESOURCES_PATH=/home/user/resources

mkdir -p $ RESOURCES_PATH

CWD=`pwd`

2. Download the hs37d5 version of the human reference genome.

cd $RESOURCES_PATH

mkdir hs37d5

cd hs37d5

wget ftp://ftp-trace.ncbi.nih.gov/1000genomes/ftp/technical/reference/phase2_reference_a

ssembly_sequence/hs37d5.fa.gz

gunzip hs37d5.fa.gz

CWD=`pwd`

***Note:*** For WES analysis, it is recommended to use the hs37d5 human genome version. It is an extension of the hg19 (GRCh37) human reference genome which contains additional sequences that have been shown to reduce the number of false positive alignments as a result of potential contaminations in WES (Li, 2014). The genome is composed of the integrated reference sequence from the GRCh37 primary assembly, comprising chromosomal plus unlocalized and unplaced contigs, the rCRS mitochondrial sequence, the genome of Human herpesvirus 4 type 1 (GenBank:NC_007605) and other concatenated decoy sequences. More details are provided within the download link and the aforementioned article.

3. Retrieve the genomic coordinates of the exome capture kit from the manufacturer as a BED file (in this case, the Agilent SureSelect All Exon v2.0 capture kit coordinates, which correspond to the data we are using from the 1000 genomes project).

cd $RESOURCES_PATH

mkdir panel

cd panel

wget --no-check-certificate https://figshare.com/ndownloader/files/33961505

mv 33961505 Agilent_SureSelect_All_Exon_V2.bed.gz

gunzip Agilent_SureSelect_All_Exon_V2.bed.gz

CWD=`pwd`

***Note:*** The timing and easiness of this step depends on the manufacturer of the capture kit, typically, instructions on how to retrieve it ship with the kit itself and should comprise no more than 5 min including booklet or online search time.

4. Download the variant annotation database files.
  a. Known variants and rs (dbSNP) accessions: dbSNP151.
  b. Further variant annotations along with variant impacts, computational pathogenicity scores, conservation scores and additional clinical information: dbNSFP 2.9.3. The latter requires a step of preprocessing for later variant annotation.
  c. Variant frequencies across population files which are required in a clinical setting to assess whether a variant has pathogenic potential according to its frequency in major population cohorts (larger frequency, less pathogenic potential): gnomAD 2.1.1 and index.

The following script template can be used to perform steps 4a–4c:

cd $RESOURCES_PATH

mkdir dbSNP

cd dbSNP

wget

ftp://ftp.ncbi.nih.gov/snp/organisms/human_9606_b151_GRCh37p13/VCF/00-

All.vcf.gz

gunzip 00-All.vcf.gz

mv 00-All.vcf dbSNP151.vcf

cd ..

mkdir dbNSFP

cd dbNSFP

wget ftp://dbnsfp:dbnsfp@dbnsfp.softgenetics.com/dbNSFPv2.9.3.zip

unzip dbNSFPv2.9.3.zip

(head -n 1 dbNSFP2.9.3_variant.chr1 ; cat dbNSFP2.9.3_variant.chr∗ | grep

-v "ˆ#") > dbNSFP2.9.3.txt

bgzip dbNSFP2.9.txt # 17’

tabix -s 1 -b 2 -e 2 dbNSFP2.9.txt.gz

cd ..

mkdir gnomAD

cd gnomAD

wget  https://storage.googleapis.com/gcp-public-data--

gnomad/release/2.1.1/vcf/exomes/gnomad.exomes.r2.1.1.sites.vcf.bgz.tbi

wget  https://storage.googleapis.com/gcp-public-data--

gnomad/release/2.1.1/vcf/exomes/gnomad.exomes.r2.1.1.sites.vcf.bgz

cd $CWD

### Prerequisite software installation – quality control

**Timing: 15 min**

The goal of this section is to download and install software required for raw data quality control. The following command line operations can be executed as provided in most Linux distributions. We are using Ubuntu 20.04 LTS. In the end of each code snippet, we include a final command which exports to the filesystem environment the command to the tool just installed. In this way, the tool usage becomes available across all the next steps. The command has the format.

export [TOOL]_PATH=path_to_the_tool/tool

where [TOOL] is the software tool just installed. Furthermore, it should be noted that certain tools and resources are versioned, meaning that the version of the file to be downloaded is subject to change. Usually, this change is evident even for the relatively inexperienced users. In the end of the section, the software required for quality control should be in the proper place for the execution of the protocol.

5. Set the directory where the tools are installed in the user’s home directory. Note that some tools, especially those dependent heavily on the Python language, may not follow this convention.

INSTALL_PATH=/home/user/tools

mkdir -p $INSTALL_PATH

CWD=`pwd`

6. Download and install FastQC.

LINK=https://www.bioinformatics.babraham.ac.uk/projects/fastqc/fastqc_v0.

11.9.zip

cd $INSTALL_PATH

wget $LINK

ARCHIVE=`basename $LINK`

unzip $ARCHIVE

export FASTQC_PATH=$INSTALL_PATH/FastQC

rm $ARCHIVE

chmod +x $FASTQC_PATH/fastqc

cd $CWD

7. Download and install MultiQC. Existence of the Python package manager pip is assumed but it is usually bundled with most current Linux systems.

pip install multiqc

export MULTIQC_PATH=/home/user/.local/bin

8. Download and install cutadapt.

pip install -–upgrade cutadapt

export CUTADAPT_PATH=/home/user/.local/bin

9. Download and install TrimGalore.

LINK=https://github.com/FelixKrueger/TrimGalore/archive/refs/tags/0.6.7.t

ar.gz

cd $INSTALL_PATH

wget $LINK -O TrimGalore_v0.6.7.tar.gz

ARCHIVE=TrimGalore_v0.6.7.tar.gz

tar -xvf $ARCHIVE

export TRIMGALORE_PATH=$INSTALL_PATH/ TrimGalore-0.6.7

rm $ARCHIVE

cd $CWD

### Prerequisite software installation – genome alignment

**Timing: 2 min**

In this section, the software required for raw read data alignment to the reference genome is retrieved and installed. At the end of the process, the software should be in the proper place for the continuation of the protocol.

10. Download and install bwa.

LINK=https://github.com/lh3/bwa/releases/download/v0.7.17/bwa-

0.7.17.tar.bz2

cd $INSTALL_PATH

wget $LINK

ARCHIVE=`basename $LINK`

tar -xvf $ARCHIVE

export BWA_PATH=$INSTALL_PATH/bwa-0.7.17

rm $ARCHIVE

cd $BWA_PATH

make

cd $CWD

### Prerequisite software installation – variant calling

**Timing: 10 min**

In this section, the software required for variant calling is retrieved and installed. At the end of the process, the software should be in the proper place for the continuation of the protocol.

11. Download and install GATK.

LINK=https://github.com/broadinstitute/gatk/releases/download/4.2.4.1/gat

k-4.2.4.1.zip

cd $INSTALL_PATH

wget $LINK

ARCHIVE=`basename $LINK`

unzip $ARCHIVE

export GATK_PATH=$INSTALL_PATH/gatk-4.2.4.1

rm $ARCHIVE

cd $CWD

12. Download and install FreeBayes.

LINK=https://github.com/freebayes/freebayes/releases/download/v1.3.6/free

bayes-1.3.6-linux-amd64-static.gz

cd $INSTALL_PATH

wget $LINK

ARCHIVE=`basename $LINK`

mkdir freebayes-1.3.6

mv $ARCHIVE ./freebayes-1.3.6/

cd freebayes-1.3.6

gunzip $ARCHIVE

chmod +x freebayes-1.3.6-linux-amd64-static

mv freebayes-1.3.6-linux-amd64-static freebayes

export FREEBAYES_PATH=$INSTALL_PATH/freebayes-1.3.6

cd $CWD

13. Download and install Docker to be able to run DeepVariant. The box below follows official instructions from here.

sudo apt remove docker docker-engine docker.io containerd runc

sudo apt update

sudo apt install -y ca-certificates curl gnupg lsb-release

curl -fsSL https://download.docker.com/linux/ubuntu/gpg | sudo gpg --

dearmor -o /usr/share/keyrings/docker-archive-keyring.gpg

echo "deb [arch=$(dpkg --print-architecture) signed-

by=/usr/share/keyrings/docker-archive-keyring.gpg]

https://download.docker.com/linux/ubuntu $(lsb_release -cs) stable" |

sudo tee /etc/apt/sources.list.d/docker.list > /dev/null

sudo apt update

sudo apt install docker-ce docker-ce-cli containerd.io

sudo usermod -aG docker ${USER}

BIN_VERSION="1.3.0"

sudo docker pull google/deepvariant:"${BIN_VERSION}"

**CRITICAL:** This is the only protocol step where the aid of a system administrator or a trained bioinformatician may be required for ensuring proper installation, as it requires system-level access.

### Prerequisite software installation – variant annotation

**Timing: 2 min**

14. Download and install SnpEff and SnpSift (in the same package).

LINK=https://snpeff.blob.core.windows.net/versions/snpEff_latest_core.zip

cd $INSTALL_PATH

wget $LINK

ARCHIVE=`basename $LINK`

unzip $ARCHIVE

export SNPEFF_PATH=$INSTALL_PATH/snpEff

rm $ARCHIVE

cd $SNPEFF_PATH

chmod +x snpEff.jar SnpSift.jar

cd $CWD

### Prerequisite software installation – generic file control and manipulation

**Timing: 30 min**

15. Download and install samtools.

LINK=https://github.com/samtools/samtools/releases/download/1.14/samtools

-1.14.tar.bz2

cd $INSTALL_PATH

wget $LINK

ARCHIVE=`basename $LINK`

tar -xvf $ARCHIVE

export SAMTOOLS_PATH=$INSTALL_PATH/samtools-1.14

rm $ARCHIVE

cd $SAMTOOLS_PATH

make

cd $CWD

16. Download and install bcftools.

LINK=https://github.com/samtools/bcftools/releases/download/1.14/bcftools

-1.14.tar.bz2

cd $INSTALL_PATH

wget $LINK

ARCHIVE=`basename $LINK`

tar -xvf $ARCHIVE

export BCFTOOLS_PATH=$INSTALL_PATH/bcftools-1.14

rm $ARCHIVE

cd $BCFTOOLS_PATH

make

cd $CWD

17. Download and install htslib.

LINK=https://github.com/samtools/htslib/releases/download/1.14/htslib-

1.14.tar.bz2

cd $INSTALL_PATH

wget $LINK

ARCHIVE=`basename $LINK`

tar -xvf $ARCHIVE

export HTSLIB_PATH=$INSTALL_PATH/htslib-1.14

rm $ARCHIVE

cd $HTSLIB_PATH

make

cd $CWD

18. Download and install bedtools.

LINK=https://github.com/arq5x/bedtools2/releases/download/v2.30.0/bedtool

s-2.30.0.tar.gz

cd $INSTALL_PATH

wget $LINK

ARCHIVE=`basename $LINK`

tar -xvf $ARCHIVE

rm $ARCHIVE

export BEDTOOLS_PATH=$INSTALL_PATH/bedtools2/bin

cd BEDTOOLS_PATH/..

make

cd $CWD

19. Download the genomic file manipulation tool library from UCSC.

cd $INSTALL_PATH

mkdir ucsc_tools

cd ucsc_tools

rsync -aP hgdownload.soe.ucsc.edu::genome/admin/exe/linux.x86_64/ ./

export UCSCTOOLS_PATH=$INSTALL_PATH/ucsc_tools

cd $CWD

20. Download and install vcflib.

LINK=https://github.com/vcflib/vcflib/releases/download/v1.0.1/vcflib-

1.0.1-src.tar.gz

cd $INSTALL_PATH

wget $LINK

ARCHIVE=`basename $LINK`

tar -xvf $ARCHIVE

rm $ARCHIVE

mv vcflib-1.0.1-src vcflib-1.0.1

export VCFLIB_PATH=$INSTALL_PATH/vcflib-1.0.1/bin

cd VCFLIB_PATH/..

make

cd $CWD

21. Download GLNexus (required for DeepVariant).

cd $INSTALL_PATH

mkdir GLnexus

cd GLnexus

wget https://github.com/dnanexus-rnd/GLnexus/releases/download/v1.4.1/glnexus_cli

chmod +x glnexus_cli

cd ..

export GLNEXUS_PATH=$INSTALL_PATH/GLnexus

cd $CWD

### Data collection

**Timing: 1.5 h**

In this section, the raw data for the demonstration of the protocol are retrieved. At the end of the process, the data should be in the proper place for the continuation of the protocol. We demonstrate the protocol using six random male-female balanced samples from the British in England and Scotland (GBR) population in the 1000 genomes project. The samples are also listed in the key resources table.

22. Set the directory where the raw data will be placed.

HOME_PATH=/home/user/analysis

FASTQ_PATH=$HOME_PATH/fastq

mkdir -p $ FASTQ_PATH

cd $CWD

23. Download the raw WES data.

cd $FASTQ_PATH

# HG00119

wget

ftp://ftp.sra.ebi.ac.uk/vol1/fastq/SRR099/SRR099967/SRR099967_1.fastq.gz

wget

ftp://ftp.sra.ebi.ac.uk/vol1/fastq/SRR099/SRR099967/SRR099967_2.fastq.gz

mv SRR099967_1.fastq.gz HG00119_1.fastq.gz

mv SRR099967_2.fastq.gz HG00119_2.fastq.gz

# HG00133

wget

ftp://ftp.sra.ebi.ac.uk/vol1/fastq/SRR099/SRR099969/SRR099969_1.fastq.gz

wget

ftp://ftp.sra.ebi.ac.uk/vol1/fastq/SRR099/SRR099969/SRR099969_2.fastq.gz

mv SRR099969_1.fastq.gz HG00133_1.fastq.gz

mv SRR099969_2.fastq.gz HG00133_2.fastq.gz

# HG00145

wget

ftp://ftp.sra.ebi.ac.uk/vol1/fastq/SRR099/SRR099957/SRR099957_1.fastq.gz

wget

ftp://ftp.sra.ebi.ac.uk/vol1/fastq/SRR099/SRR099957/SRR099957_2.fastq.gz

mv SRR099957_1.fastq.gz HG00145_1.fastq.gz

mv SRR099957_2.fastq.gz HG00145_2.fastq.gz

# HG00239

wget

ftp://ftp.sra.ebi.ac.uk/vol1/fastq/SRR099/SRR099958/SRR099958_1.fastq.gz

wget

ftp://ftp.sra.ebi.ac.uk/vol1/fastq/SRR099/SRR099958/SRR099958_2.fastq.gz

mv SRR099958_1.fastq.gz HG00239_1.fastq.gz

mv SRR099958_2.fastq.gz HG00239_2.fastq.gz

# HG00258

wget

ftp://ftp.sra.ebi.ac.uk/vol1/fastq/SRR099/SRR099954/SRR099954_1.fastq.gz

wget

ftp://ftp.sra.ebi.ac.uk/vol1/fastq/SRR099/SRR099954/SRR099954_2.fastq.gz

mv SRR099954_1.fastq.gz HG00258_1.fastq.gz

mv SRR099954_2.fastq.gz HG00258_2.fastq.gz

# HG00265

wget

ftp://ftp.sra.ebi.ac.uk/vol1/fastq/SRR099/SRR099968/SRR099968_1.fastq.gz

wget

ftp://ftp.sra.ebi.ac.uk/vol1/fastq/SRR099/SRR099968/SRR099968_2.fastq.gz

mv SRR099968_1.fastq.gz HG00265_1.fastq.gz

mv SRR099968_1.fastq.gz HG00265_2.fastq.gz

cd $CWD

## Key resources table

**Table:** 

REAGENT or RESOURCE	SOURCE	IDENTIFIER
**Deposited data**		

HG00119 (Male)	1000 genomes GBR	https://www.ncbi.nlm.nih.gov/sra/SRR099967
HG00133 (Female)	1000 genomes GBR	https://www.ncbi.nlm.nih.gov/sra/SRR099969
HG00145 (Male)	1000 genomes GBR	https://www.ncbi.nlm.nih.gov/sra/SRR099957
HG00239 (Female)	1000 genomes GBR	https://www.ncbi.nlm.nih.gov/sra/SRR099958
HG00258 (Female)	1000 genomes GBR	https://www.ncbi.nlm.nih.gov/sra/SRR099954
HG00265 (Male)	1000 genomes GBR	https://www.ncbi.nlm.nih.gov/sra/SRR099968
SureSelect AllExon 2.0	Agilent	https://doi.org/10.6084/m9.figshare.19115102
Human genome hs37d5	1000 genomes consortium	ftp://ftp-trace.ncbi.nih.gov/1000genomes/ftp/technical/reference/phase2_reference_assembly_sequence/hs37d5.fa.gz
dbSNP 151	NCBI, (Sherry et al., 1999)	ftp://ftp.ncbi.nih.gov/snp/organisms/human_9606_b151_GRCh37p13/VCF/00-All.vcf.gz
dbNSFP	(Liu et al., 2016)	ftp://dbnsfp:dbnsfp@dbnsfp.softgenetics.com/dbNSFPv2.9.3.zip
gnomAD	gnomAD consortium, (Karczewski et al., 2020)	https://storage.googleapis.com/gnomad-public/release/2.1.1/vcf/exomes/gnomad.exomes.r2.1.1.sites.vcf.bgz

**Software and algorithms**		

FastQC	https://www.bioinformatics.babraham.ac.uk/projects/fastqc/	https://github.com/s-andrews/FastQC
MultiQC	(Ewels et al., 2016)	https://multiqc.info/
Cutadapt	(Martin, 2011)	https://cutadapt.readthedocs.io/
Trim Galore!	https://www.bioinformatics.babraham.ac.uk/projects/trim_galore/	https://github.com/FelixKrueger/TrimGalore
bwa	(Li and Durbin, 2009)	https://github.com/lh3/bwa
GATK	(DePristo et al., 2011)	https://github.com/broadinstitute/gatk
FreeBayes	(Garrison and Marth, 2012)	https://github.com/freebayes/freebayes
DeepVariant	(Poplin et al., 2018)	https://github.com/google/deepvariant
SnpEff	(Cingolani et al., 2012)	https://pcingola.github.io/SnpEff/
samtools	(Danecek et al., 2021)	http://www.htslib.org/
bcftools	(Danecek et al., 2021)	http://www.htslib.org/
htslib	(Bonfield et al., 2021)	http://www.htslib.org/
BEDTools	(Quinlan and Hall, 2010)	https://bedtools.readthedocs.io/
UCSC tools	(Kuhn et al., 2013)	http://hgdownload.soe.ucsc.edu/admin/exe/
vcflib	(Garrison and Marth, 2012)	https://github.com/vcflib/vcflib
GLnexus	(Lin et al., 2018)	https://github.com/dnanexus-rnd/GLnexus
R	(Ihaka and Gentleman, 1996)	https://www.r-project.org/

**Other**		

Recommended hardware: - 16 physical core system - 128 GB of RAM - Ubuntu 18.04 operating system	N/A	N/A

## Materials and equipment

Hardware: while setting up the computational protocol, the steps were performed in a 64 physical core system with 512 GB of RAM and Ubuntu 20.04 LTS. We used 32 cores where parallelization was available. Generally, the process can be completed with adequate performance in a system with 16 cores and 128 GB of RAM. If less RAM is available, parallelization can be avoided partly by restricting the number of jobs executed asynchronously in the background (remove the ‘&’ character where it is found in several commands).

## Step-by-step method details

In all the subsequent steps, the paths to the required software tools are the same as the “exported” paths in the respective command boxes under the “before you begin” section.

### Quality control and filtering

**Timing: 2 h 15 min**

Quality control of the generated data is a crucial step in every Next Generation Sequencing protocol, let alone in the case of processes related also to the clinic, such as exome sequencing and variant calling. Quality control in exomes becomes even more critical, as in the case of detecting variants on a large scale, it is not straightforward to distinguish between sequencing errors and actual variations in the human genome. Therefore, quality control procedures are often lenient and total quality assessment is a combination of various factors. In this section we outline a typical pre-alignment quality control procedure for whole exome sequencing data. In the end, quality controlled FASTQ files ready for alignment will be acquired.

1. Quality control with FastQC and MultiQC.
  a. Pre-alignment QC using FastQC to determine if any raw data corrective actions need to be taken. Default FastQC reports are not interactive and not aggregated.
  b. Use MultiQC to create a more user-friendly and complete report.

The following bash script can be used as a template:

#!/bin/bash

HOME_PATH=/home/user/analysis

FASTQ_PATH=$HOME_PATH/fastq

FASTQ_PATTERN=∗.fastq.gz

FASTQC_COMMAND=$FASTQC_PATH/fastqc

MULTIQC_COMMAND=$MULTIQC_PATH/multiqc

FASTQC_OUTPUT=$HOME_PATH/fastqc

CORES=8

if [ ! -d $FASTQC_OUTPUT ]

then

 mkdir -p $FASTQC_OUTPUT

fi

$FASTQC_COMMAND --outdir $FASTQC_OUTPUT --threads $CORES

$FASTQ_PATH/$FASTQ_PATTERN

$MULTIQC_COMMAND $FASTQC_OUTPUT -o $FASTQC_OUTPUT

The results of MultiQC can be viewed by opening the file *$FASTQC_OUTPUT/multiqc_report.html* in a web browser.

***Note:*** From the results of FastQC and MultiQC, a lot of useful information may be revealed. Some examples include the presence of adapters, the presence of bias in the 3′/5′ end of reads, poor quality in the 3′/5′ end of reads, poor quality for certain samples and sequence over-representation other than the one expected from adapter contamination. After a first round of inspection, we may have to improve the quality of the overall dataset prior to continuing with other actions regarding alignment to the reference genome and the subsequent variant calling. Trim Galore is a good option for this as it automates many processes, including standard adapter automated removal and maintaining paired-end read integrity. In the case of the data presented in this protocol, the quality of the dataset is acceptable and none of the above points apply. No further further action is needed. Therefore, the following section is *not* required. It is only mentioned here for reference purposes and protocol completeness.

2. Adapter and poor-quality base trimming (optional). A template bash script to wrap Trim Galore follows. With comments, below the main command, a stricter alternative filtering approach:

#!/bin/bash

HOME_PATH=/PATH/TO/ANALYSIS/DIRECTORY

FASTQ_PATH=$HOME_PATH/fastq

TRIMGALORE_COMMAND=$TRIMGALORE_PATH/trim_galore

CUTADAPT_COMMAND=$CUTADAPT_PATH/cutadapt

TRIMGALORE_OUTPUT=$HOME_PATH/fastq_qual

CORES=4

if [ ! -d $TRIMGALORE_OUTPUT ]

then

 mkdir -p $TRIMGALORE_OUTPUT

fi

for FILE in $FASTQ_PATH/∗_1.fastq.gz

do

 BASE=`basename $FILE | sed s/_1\.fastq\.gz//`

 echo "Processing $BASE"

 mkdir -p $TRIMGALORE_OUTPUT

 F1=$FASTQ_PATH/$BASE"_1.fastq.gz"

 F2=$FASTQ_PATH/$BASE"_2.fastq.gz"

  $TRIMGALORE_COMMAND \

  --quality 30 \

  --length 50 \

  --output_dir $TRIMGALORE_OUTPUT/$BASE \

  --path_to_cutadapt $CUTADAPT_COMMAND \

  --cores 4 \

  --paired \

  --fastqc \

  --trim-n $F1 $F2

 mv $TRIMGALORE_OUTPUT/$BASE"_1_val_1.fq.gz" \

 $TRIMGALORE_OUTPUT/$BASE"_1.fastq.gz"

 mv $TRIMGALORE_OUTPUT/$BASE"_2_val_2.fq.gz" \

 $TRIMGALORE_OUTPUT/$BASE"_2.fastq.gz"

 mv $TRIMGALORE_OUTPUT/$BASE"_1_val_1_fastqc.html" \

 $TRIMGALORE_OUTPUT/$BASE"_1_fastqc.html"

 mv $TRIMGALORE_OUTPUT/$BASE"_1_val_1_fastqc.zip" \

 $TRIMGALORE_OUTPUT/$BASE"_1_fastqc.zip"

 mv $TRIMGALORE_OUTPUT/$BASE"_2_val_2_fastqc.html" \

 $TRIMGALORE_OUTPUT/$BASE"_2_fastqc.html"

 mv $TRIMGALORE_OUTPUT/$BASE"_2_val_2_fastqc.zip" \

 $TRIMGALORE_OUTPUT/$BASE"_2_fastqc.zip"

done

For paired-end reads, Trim Galore! produces four outputs and specifically, mate 1 reads passing QC, mate 2 reads passing QC (and matched to mate 1), mate 1 failed reads (optional, not chosen above), mate 2 failed reads (optional, not chosen above).

3. Inspection of the outcome.
  Trim Galore also runs FastQC again. From its output we may be able to see that:
  a. The problematic points identified above are remedied and brought to acceptable states and error rates.
  b. The number of filtered reads remains at acceptable amounts.

### Alignment to the reference genome and alignment statistics

**Timing: 4 h 30 min**

This section describes the process of aligning the FASTQ pairs to the reference genome and collecting alignment statistics for quality control purposes. In the end of the step, BAM files and a report of read alignment statistics are generated.

4. Index the reference genome.

This step is needed only once and does not have to be repeated for the application of the protocol to new data, unless the index and/or reference genomes are deleted by the user. When this process is completed, we need to create an additional file called hs37d5.dict expected by GATK tools for variant calling and other processing. We use samtools for this.

cd $RESOURCES_PATH/hs37d5

$BWA_PATH/bwa index hs37d5.fa

$SAMTOOLS_PATH/samtools faidx hs37d5.fa

$SAMTOOLS_PATH/samtools dict hs37d5.fa > hs37d5.dict

cd $CWD

5. Alignment to the reference genome.

After the index building is finished, the alignment process can be initiated for each FASTQ file.

**CRITICAL:** The downstream variant calling analysis requires read group information. Read groups are added to each alignment resulting in a BAM file in order to separate different individuals as well as samples resulting from different lanes and libraries. Read groups are required as variant callers pool samples to estimate the models behind variant discovery. Read groups (the RG tag) can be added during alignment with bwa using the -R option. The following shell script can be used to accomplish the alignment and read group addition procedure. Furthermore, as BAM files need further processing, the file extension of the aligned files is .uns.

#!/bin/bash

HOME_PATH=/home/user/analysis

# Change the path below with the quality-controlled data directory

# if trimming performed (see commented line below)

FASTQ_PATH=$HOME_PATH/fastq

#FASTQ_PATH=$HOME_PATH/fastq_qual

BAM_PATH=$HOME_PATH/bam

THREADS=24

BWA_INDEX=$RESOURCES_PATH/hs37d5/hs37d5.fa

if [ -d $BAM_PATH ]

then

 mkdir -p $BAM_PATH

fi

for FILE in `ls $FASTQ_PATH/∗_1.fastq.gz`

do

  BASE=`basename $FILE | sed s/_1\.fastq\.gz//`

  F1=$FASTQ_PATH/$BASE"_1.fastq.gz"

  F2=$FASTQ_PATH/$BASE"_2.fastq.gz"

  RG="@RG\tID:"$BASE"\tSM:"$BASE"\tLB:WES\tPL:ILLUMINA"

  $BWA_PATH/bwa mem -t $THREADS -R $RG $BWA_INDEX $F1 $F2 | \

    $SAMTOOLS_PATH/samtools view -bS -o $BAM_PATH/$BASE".uns" -

done

### Preparation of BAM files

**Timing: 3 h**

In this section we describe the steps taken to prepare the BAM files for the subsequent variant calling and discovery. The output of this part comprises BAM files suitable for the subsequent variant calling. The vast majority of variant callers require these preparation steps and the major steps taken (in slightly different flavors according to the tools used to make them so) are the following:

6. Merging of BAM files from different lanes. This is an optional step according to the instrument and sequencing protocol used (the files used in this protocol do not require this step).
7. Then, if the sequencing is paired-end:
  a. Sort the reads in the BAM file according to their names so that pairs are placed one below the other.
  b. Fix mates so that they both have the same sets of attributes for the subsequent preprocessing.
  c. Re-sort the reads according to their genomic coordinates this time.
  d. Mark the duplicate reads as variant callers take this information into account.
8. If the sequencing is single-end:
  a. Sort the reads according to their genomic coordinates.
  b. Mark the duplicate reads as variant callers take this information into account.

In our case, we have paired-end sequencing, so we are following the first set of steps above.

9. Sort the reads in the BAM file according to their names so that pairs are placed one below the other and fix read-mates so that they both have the same sets of attributes for the subsequent preprocessing.

#!/bin/bash

BAM_PATH=$HOME_PATH/bam

CORES=16

for FILE in `ls $BAM_PATH/∗.uns`

do

 SAMPLE=`basename $FILE | sed s/\.uns//`

 echo "Processing $SAMPLE"

 $SAMTOOLS_PATH/samtools sort -n -@ $CORES -m 4G \

  $BAM_PATH/$SAMPLE".uns" | \

  $SAMTOOLS_PATH/samtools fixmate -m -

  $BAM_PATH/$SAMPLE"_fixmate.bam"

done

rm $BAM_PATH/∗.uns

10. Re-sort the reads according to their genomic coordinates and mark the duplicate reads as variant callers take this information into account.

#!/bin/bash

BAM_PATH=$HOME_PATH/bam

CORES=16

for FILE in `ls $BAM_PATH/∗_fixmate.bam`

do

 SAMPLE=`basename $FILE | sed s/_fixmate\.bam//`

 echo "Processing $SAMPLE"

 $SAMTOOLS_PATH/samtools sort -@ $CORES -m 4G \

  $BAM_PATH/$SAMPLE"_fixmate.bam" | \

  $SAMTOOLS_PATH/samtools markdup - $BAM_PATH/$SAMPLE".bam"

 echo "Indexing $SAMPLE"

 $SAMTOOLS_PATH/samtools index $BAM_PATH/$SAMPLE".bam"

done

### Collection of alignment statistics

**Timing: 3 h 15 min**

In this section, several statistics related the quality control of the alignment process are collected. At the end of the process, a text file with statistics should be produced.

11. Collect alignment statistics for quality control.
  a. Total sequenced reads.
  b. Aligned reads.
  c. Uniquely aligned reads (q>20).
  d. Chimeric reads (for paired-end sequencing).
  e. Reads overlapping targets.
  f. Total sequenced bases.
  g. Aligned bases.
  h. Uniquely aligned bases.
  i. Bases overlapping targets.
    Furthermore, for paired-end sequencing, we collect:
  j. Total sequenced read pairs.
  k. Properly aligned read pairs.
  l. Properly paired uniquely aligned reads.

The following shell script template can be used for this purpose:

#!/bin/bash

CAPTURE_KIT=$HOME_PATH/resources/panel/Agilent_SureSelect_All_Exon_V2.bed

BAM_PATH=$HOME_PATH/bam

REPORT=$HOME_PATH/reports/finalbamstats.txt

mkdir $HOME_PATH/reports

printf "%s\t%s\t%s\t%s\t%s\t%s%s\t%s\t%s\t%s\t%s\t%s\t%s\n" "name" \

 "total reads" "total reads pairs" "aligned reads" \

  "properly paired aligned pairs" "uniquely aligned reads (q>20)" \

  "properly paired uniquely aligned reads" "chimeric reads" \

    "reads overlapping targets" "total bases" "aligned bases" \

    "uniquely aligned bases" "bases overlapping targets" > $REPORT

for FILE in `ls $BAM_PATH/∗_fixmate.bam`

do

 SAMPLE=`basename $FILE | sed s/_fixmate\.bam//`

 echo "Processing $SAMPLE"

 BAM=$BAM_PATH/$SAMPLE".bam"

 printf "%s\t" $SAMPLE >> $REPORT

 echo " total reads"

 printf "%d\t" `$SAMTOOLS_PATH/samtools view -c -F2048 $BAM` >> $REPORT

 echo " total read pairs"

 printf "%d\t" `$SAMTOOLS_PATH/samtools view -c -F2048 $BAM | awk '{print $1/2}'` \

  >> $REPORT

 echo " aligned reads"

 printf "%d\t" `$SAMTOOLS_PATH/samtools view -c -F2052 $BAM` >> $REPORT

 echo " properly paired aligned pairs"

 printf "%d\t" `$SAMTOOLS_PATH/samtools view -c -f66 -F2048 $BAM` \

  >> $REPORT

 echo " uniquely aligned reads (q>20)"

 printf "%d\t" `$SAMTOOLS_PATH/samtools view -c -F2052 -q20 $BAM` >> \

  $REPORT

 echo " properly paired uniquely aligned reads"

 printf "%d\t" `$SAMTOOLS_PATH/samtools view -c -f66 -F2048 -q20 $BAM` \

  >> $REPORT

 echo " chimeric reads"

 printf "%d\t" `

  $SAMTOOLS_PATH/samtools flagstat $BAM | \

  perl -e 'my @in;' \

   -e 'while(<>) { chomp $_; push(@in,$_); }' \

   -e 'my @tmp = split("\\\+",pop(@in));' \

   -e '$tmp[0] =∼ s/\s+$//;' \

   -e 'print STDOUT $tmp[0];'

  ` >> $REPORT

 echo " reads overlapping targets"

 printf "%d\t" `

  $BEDTOOLS_PATH/bedtools intersect -a $CAPTURE_KIT -b $BAM -c | \

    awk 'BEGIN {tot=0}{tot+=$4} END {print tot}'

  ` >> $REPORT

 echo " total bases"

 printf "%d\t" `

  $SAMTOOLS_PATH/samtools view $BAM | cut -f10 | \

    awk 'BEGIN {tr=0}{tr+=length($0)} END {print tr}'

  ` >> $REPORT

 echo " aligned bases"

 printf "%d\t" `

  $SAMTOOLS_PATH/samtools view -F2052 $BAM | cut -f10 | \

    awk 'BEGIN {tr=0}{tr+=length($0)} END {print tr}'

  ` >> $REPORT

 echo " uniquely aligned bases"

 printf "%d\t" `

  $SAMTOOLS_PATH/samtools view -F2052 -q20 $BAM | cut -f10 | \

    awk 'BEGIN {tr=0}{tr+=length($0)} END {print tr}'

  ` >> $REPORT

 echo " bases overlapping targets"

 printf "%d\n" `

  $BEDTOOLS_PATH/bedtools coverage -a $CAPTURE_KIT -b $BAM -d | \

    awk 'BEGIN {tr=0} {tr+=$5} END {print tr}'

  ` >> $REPORT

done

***Note:*** This section describes the steps taken to collect some useful alignment statistics and prepare the BAM files for the subsequent variant calling and discovery. The former may further help identify poor quality samples that should not be used for variant calling. While such samples may have passed the QC process applied on raw data, it is possible that they may present low alignment rates or low coverage over the target areas (exome capture kit), as for example a result of possible contamination.

### Signal visualization

**Timing: 30 min**

Another level of quality control as well as supporting evidence for discovered variants is the actual inspection of the sequencing signal or coverage (i.e., the histogram created by the short reads pileup in a specific locus). This can be accomplished by uploading, opening or linking signal files created from BAM files, to a genome browser such as the UCSC Genome Browser or the IGV (Robinson et al., 2011). Signal tracks in BigWig format can be created using the following shell script as a template. In this case, we note the addition of the “chr” short string before the chromosome names. This is required for viewing in the UCSC Genome Browser. For other browsers such as IGV, this addition depends on the reference genome loaded. The latter can be controlled in IGV but not in the UCSC Genome Browser. The output of this part is BigWig files suitable for visualization in a genome browser.

#!/bin/bash

BAM_PATH=$HOME_PATH/bam

TRACKS_PATH=$HOME_PATH/tracks

GENOME_SIZE=$BEDTOOLS_PATH/../genomes/human.hg19.genome

if [ -d $TRACKS_PATH ]

then

 mkdir -p $TRACKS_PATH

fi

for FILE in `ls $BAM_PATH/∗_fixmate.bam`

do

 SAMPLE=`basename $FILE | sed s/_fixmate\.bam//`

 echo "Processing $SAMPLE"

 $BEDTOOLS_PATH/bedtools genomecov -bg \

   -ibam $BAM_PATH/$SAMPLE/$SAMPLE".bam" | \

   grep -vP 'chrU|rand|hap|loc|cox|GL|NC|hs37d5' | \

   awk '{print "chr"$1"\t"$2"\t"$3"\t"$4}' | \

   sed s/chrMT/chrM/g | \

   sort -k1,1 -k2g,2 > $TRACKS_PATH/$SAMPLE".bedGraph" &

done

wait

for FILE in `ls $TRACKS_PATH/∗.bedGraph`

do

 echo "Processing $FILE"

 SAMPLE=`basename $FILE | sed s/\.bedGraph//`

 $UCSCTOOLS_PATH/bedGraphToBigWig $FILE $GENOME_SIZE

$TRACKS_PATH/$SAMPLE".bigWig" &

done

wait

rm $TRACKS_PATH/∗.bedGraph

The produced BigWig files must then either be put in a directory served by a web browser such as Apache in order to be viewed by a web-based genome browser (such as UCSC) or be opened directly in a local genome browser such as IGV.

### Variant calling with GATK HaplotypeCaller

**Timing: 6 h**

This section describes the variant calling procedure using GATK HaplotypeCaller and its output is a VCF file with filtered variants after the application of basic filters. Each caller accepts the BAM files as main inputs but in order to be as efficient as possible, different pre-calling procedures are required. Examples of such procedures are:

Example 1: The GATK HaplotypeCaller requires a procedure called Base Quality Score Recalibration (BQSR) in order for its underlying model to work as best as possible.

Example 2: For parallel execution, HaplotypeCaller and FreeBayes require the splitting of the capture kit target genomic intervals so that the algorithm operates on different intervals in parallel. However, the capture kit should be split using different strategies for each caller.

Example 3: DeepVariant on the other hand does the splitting of the capture kit regions automatically.

In addition, there is nowadays some debate on whether BQSR is needed prior to variant calling or not, as this process was initially developed for older sequencers that produced poorer results than modern ones. We choose to apply BQSR for protocol completeness purposes. More info on the debate can be found in the official GATK community forums and other bioinformatics communities such as Biostars.

The calling process with GATK HaplotypeCaller has several steps and substeps. Below we outline the process and provide template scripts.

12. Base Quality Score Recalibration and application on BAM files.
  a. Split the capture kit to as many intervals as the cores we wish to use.
  b. Calculate separate BQSR reports.
  c. Gather these reports to a joint model.
  d. Apply the model to existing BAM files.
  e. Keep the original BAM files as they are required unchanged by the other variant callers.

The following shell script template can be used for BQSR:

#!/bin/bash

BAM_PATH=$HOME_PATH/bam

CAPTURE_KIT=$RESOURCES_PATH/panel/Agilent_SureSelect_All_Exon_V2.bed

INTERVAL_LIST_PATH=$HOME_PATH/resources/interval_scatter

BWA_INDEX=$RESOURCES_PATH/hs37d5/hs37d5.fa

DBSNP=$RESOURCES_PATH/dbSNP151.vcf

GNOMAD=$RESOURCES_PATH/gnomad.exomes.r2.1.1.sites.vcf.bgz

CORES=16

PADDING=50

# Process dbSNP

$HTSLIB_PATH/bgzip $DBSNP

$HTSLIB_PATH/tabix $DBSNP”.gz”

DBSNP=$RESOURCES_PATH/dbSNP151.vcf.gz

mkdir -p $HOME_PATH/reports

META_REPORT=$HOME_PATH/reports/bsqr_current.log

echo "=== Splitting intervals" > $META_REPORT

if [ -d $INTERVAL_LIST_PATH ]

then

 echo " Cleaning previous intervals" >> $META_REPORT

 rm -r $INTERVAL_LIST_PATH

fi

mkdir -p $INTERVAL_LIST_PATH

# Firstly split exome intervals for parallel BSQR

$GATK_PATH/gatk SplitIntervals \

  --reference $BWA_INDEX \

  --intervals $CAPTURE_KIT \

  --interval-padding $PADDING \

  --scatter-count $CORES \

  --output $INTERVAL_LIST_PATH \

  --QUIET

echo "=== Calculating BQSR tables" >> $META_REPORT

for FILE in `ls $BAM_PATH/∗_fixmate.bam`

do

  SAMPLE=`basename $FILE | sed s/_fixmate\.bam//`

  echo "Processing $SAMPLE" >> $META_REPORT

  BAM=$BAM_PATH/$SAMPLE/$SAMPLE".bam"

  mkdir -p $BAM_PATH/$SAMPLE

  BQSR_PART_OUT=$BAM_PATH/$SAMPLE/bqsr_parts

  if [ -d $BQSR_PART_OUT ]

  then

   echo " Cleaning previous tables" >> $META_REPORT

   rm -r $BQSR_PART_OUT

  fi

  mkdir -p $BQSR_PART_OUT

  # Calculate BQSR over intervals

  for INTERVAL in `readlink -f $INTERVAL_LIST_PATH/∗`

  do

    BQSR_NAME=`basename $INTERVAL | sed s/\-scattered\.interval_list//`

    echo " Processing $BQSR_NAME" >> $META_REPORT

    $GATK_PATH/gatk BaseRecalibrator \

     --input $BAM \

     --reference $BWA_INDEX \

     --output $BQSR_PART_OUT/$BQSR_NAME".tab" \

     --known-sites $DBSNP \

     --known-sites $GNOMAD \

     --intervals $INTERVAL \

     --interval-padding $PADDING \

     --QUIET &

  done

  # Wait for individuals to complete before moving to the next thread

  wait

done

echo "=== Gathering BQSR reports" >> $META_REPORT

for FILE in `ls $BAM_PATH/∗_fixmate.bam`

do

  SAMPLE=`basename $FILE | sed s/_fixmate\.bam//`

  echo "Processing reports for $SAMPLE" >> $META_REPORT

  BQSR_PART_OUT=$BAM_PATH/$SAMPLE/bqsr_parts

  for TAB in `readlink -f $BQSR_PART_OUT/∗`

  do

   echo "--input $TAB" >> $BAM_PATH/$SAMPLE/gather_bqsr.arg

  done

  # Gather reports

  $GATK_PATH/gatk GatherBQSRReports \

    --arguments_file $BAM_PATH/$SAMPLE/gather_bqsr.arg \

    --output $BAM_PATH/$SAMPLE/bqsr.tab \

    --QUIET &

done

# Wait for BQSR tables to be merged for each sample

wait

echo "=== Applying BQSR to BAM files" >> $META_REPORT

for FILE in `ls $BAM_PATH`

do

  SAMPLE=`basename $FILE | sed s/_fixmate\.bam//`

  echo "Processing BAM file $SAMPLE" >> $META_REPORT

  BAM=$BAM_PATH/$SAMPLE/$SAMPLE".bam"

  BQSR_TABLE=$BAM_PATH/$SAMPLE/bqsr.tab

  # Apply BQSR to BAM files

  $GATK_PATH/gatk ApplyBQSR \

    --input $BAM \

    --reference $BWA_INDEX \

    --bqsr-recal-file $BQSR_TABLE \

    --output $BAM_PATH/$SAMPLE/$SAMPLE"_bqsr.bam" \

    --QUIET &

done

# Wait for new BAM files to be created before reporting finished

wait

echo "=== Finished!" >> $META_REPORT

***Note:*** BQSR is a relatively lengthy process and can be executed in parallel if we split the capture kit genomic regions. The GATK toolkit has tools for this. The main inputs for BQSR in exome sequencing are, the exome capture kit, the reference genome and a list of known variant locations (e.g., dbSNP, gnomAD) used to provide the algorithm with a list of ground truth sites used to recalibrate scores.

After the BQSR process, we are ready to proceed with variant calling for each sample separately. Although there are many alternatives to run exome analysis with HaplotypeCaller in an efficient way (e.g., parallelization of capture intervals or running each sample on the background or even using GNU parallel), we propose the following sub-protocol (“intervals” are the capture kit genomic intervals created during the BQSR process).

13. Base Quality Score Recalibration and application on BAM files.
  a. For each sample.
    i. For each genomic interval use GATK HaplotypeCaller to create a gVCF callset file. The files for each interval are written in a sample-specific directory.
  b. For each sample.
    i. Loop through created gVCFs and create a list file.
    ii. Merge gVCFs by placing one below the other and create one unique gVCF file.
    iii. For each sample, sort the consolidated gVCF using GATK SortVcf.
  c. Create a list file for input to GATK GenotypeGVCFs.
  d. Call GATK GenotypeGVCFs to create the final callset in VCF format.
  e. Using bcftools.
  f. Apply the GATK best hard filtering practices for SNPs and create a filtered SNP VCF.
    i. Apply the GATK best hard filtering practices for INDELs and create a filtered INDEL VCF while at the same time normalizing the INDELs.
    ii. With the SNP and INDEL filtered VCFs, use GATK MergeVcfs to merge the separate filtered VCF files.
  g. Cleanup.

**CRITICAL:** At this point and with respect to step 2e above, it should be noted that the best filtering practices suggested by the GATK community comprise only basic variant filters in order to reduce noise. As with the rest of the variant callers, more elaborate filtering should follow, which is not generic as these filters but it is application dependent. For example, a user investigating rare disease should look for damaging variants (e.g., frameshift, splicing, missense) after variant annotation while a user interested in conducting a population study with many samples should focus on filtering variants with low frequencies as those would not characterize a population cohort. Finally, under different clinical settings, a user would possibly combine various filters, for example restrict damaging variants to certain virtual gene panels of interest.

The suggested hard filters by the GATK community for multiple samples are:

For SNPs: QD < 2.0, MQ < 40.0, FS > 60.0, SOR > 3.0, MQRankSum < -12.5, ReadPosRankSum < -8.0.

For Indels: QD < 2.0, ReadPosRankSum < -20.0, InbreedingCoeff < -0.8, FS > 200.0, SOR > 10.0.

Summaries for all steps (including background processes) are recorded in a “report” file for general supervision. The following shell script template can be used to run the above steps:

#!/bin/bash

export VCF_PATH=$HOME_PATH/vcf

BAM_PATH=$HOME_PATH/bam

INTERVAL_LIST_PATH=$RESOURCES_PATH/panel/interval_scatter

BWA_INDEX=$RESOURCES_PATH/hs37d5/hs37d5.fa

CORES=16

PADDING=50

META_REPORT=$HOME_PATH/reports/haca_current.log

echo "=== Calling variants" > $META_REPORT

for SAMPLE in `ls $BAM_PATH`

do

 echo "Processing $SAMPLE" >> $META_REPORT

 BAM=$BAM_PATH/$SAMPLE/$SAMPLE"_bqsr.bam"

 GVCF_PART_OUT=$BAM_PATH/$SAMPLE/gvcf_parts

 if [ -d $GVCF_PART_OUT ]

 then

  echo " Cleaning previous gVCFs" >> $META_REPORT

  rm -r $GVCF_PART_OUT

 fi

 mkdir -p $GVCF_PART_OUT

 # Call variants over intervals

 for INTERVAL in `readlink -f $INTERVAL_LIST_PATH/∗`

 do

  GVCF_NAME=`basename $INTERVAL | sed s/\-scattered\.interval_list//`

  echo " Processing $GVCF_NAME" >> $META_REPORT

  $GATK_PATH/gatk HaplotypeCaller \

    --input $BAM \

    --reference $BWA_INDEX \

    --intervals $INTERVAL \

    --interval-padding $PADDING \

    --output $GVCF_PART_OUT/$GVCF_NAME".g.vcf" \

    --emit-ref-confidence GVCF \

    --create-output-variant-index false \

    --QUIET &

 done

 # Wait for individuals to complete before moving to the next thread

 wait

done

# Then GVCFs must be consolidated

echo "=== Merging gVCFs" >> $META_REPORT

for SAMPLE in `ls $BAM_PATH`

do

 echo "Processing interval gVCFs for $SAMPLE"

 GVCF_PART_OUT=$BAM_PATH/$SAMPLE/gvcf_parts

 if [ -f $BAM_PATH/$SAMPLE/interval_gvcfs.txt ]

 then

  echo " Cleaning previous gVCFs input file" >> $META_REPORT

  rm $BAM_PATH/$SAMPLE/interval_gvcfs.txt

 fi

 for GVCF in `readlink -f $GVCF_PART_OUT/∗.g.vcf`

 do

  echo "$GVCF" >> $BAM_PATH/$SAMPLE/interval_gvcfs.txt

 done

 # Get the gVCF header and strip the GATK command

 GVFH=`readlink -f $GVCF_PART_OUT/∗.g.vcf | head -1`

 grep "ˆ#" $GVFH | grep -v "ˆ##GATKCommand" > $BAM_PATH/$SAMPLE/gvcf.header

 # Cat the gVCFs

 for GVCF in `readlink -f $GVCF_PART_OUT/∗.g.vcf`

 do

  echo " Concatenating $GVCF"

  #echo " Concatenating $GVCF" >> $META_REPORT

 grep -v "ˆ#" $GVCF >> $BAM_PATH/$SAMPLE/gvcf.tmp

 done

 # Place the header

 echo " Creating final gVCF"

 #echo " Creating final gVCF" >> $META_REPORT

 cat $BAM_PATH/$SAMPLE/gvcf.header $BAM_PATH/$SAMPLE/gvcf.tmp > \

  $BAM_PATH/$SAMPLE/$SAMPLE".u.g.vcf"

 rm $BAM_PATH/$SAMPLE/gvcf.tmp $BAM_PATH/$SAMPLE/gvcf.header

done

# Sort gVCFs

echo "=== Sorting gVCFs" >> $META_REPORT

for SAMPLE in `ls $BAM_PATH`

do

 echo "Sorting gVCF for $SAMPLE" >> $META_REPORT

 $GATK_PATH/gatk SortVcf \

   --INPUT $BAM_PATH/$SAMPLE/$SAMPLE".u.g.vcf" \

   --OUTPUT $BAM_PATH/$SAMPLE/$SAMPLE".g.vcf.gz" \

   --QUIET &

done

# Wait for sorting to finish before cleaning unsorted

wait

# Some cleanup

echo "=== Deleting unsorted gVCFs" >> $META_REPORT

for FILE in `ls $BAM_PATH/∗_fixmate.bam`

do

 SAMPLE=`basename $FILE | sed s/_fixmate\.bam//`

 echo "Deleting unsorted gVCF for $SAMPLE" >> $META_REPORT

 rm $BAM_PATH/$SAMPLE/$SAMPLE".u.g.vcf"

 echo "Compression gVCF parts for $SAMPLE" >> $META_REPORT

 pigz $BAM_PATH/$SAMPLE/gvcf_parts/∗

 echo "Compression BQSR reports for $SAMPLE" >> $META_REPORT

 pigz $BAM_PATH/$SAMPLE/bqsr_parts/∗

done

# Gather VCFs

echo "=== Combining sorted population gVCFs" >> $META_REPORT

if [ ! -d $VCF_PATH ]

then

 mkdir $VCF_PATH

fi

# Delete the .arg file as it will get multiple entries

if [ -f $VCF_PATH/combine_gvcf.arg ]

then

 rm $VCF_PATH/combine_gvcf.arg

fi

for FILE in `ls $BAM_PATH/∗_fixmate.bam`

do

 SAMPLE=`basename $FILE | sed s/_fixmate\.bam//`

 GVCF=`readlink -f $BAM_PATH/$SAMPLE/$SAMPLE".g.vcf.gz"`

 echo "--variant $GVCF" >> $VCF_PATH/combine_gvcf.arg

done

# Combine gVCFs

$GATK_PATH/gatk CombineGVCFs \

 --reference $BWA_INDEX \

 --arguments_file $VCF_PATH/combine_gvcf.arg \

 --output $VCF_PATH/haplotypecaller_full.g.vcf.gz

# Genotype VCFs

echo "=== Genotyping gVCFs" >> $META_REPORT

$GATK_PATH/gatk GenotypeGVCFs \

  --reference $BWA_INDEX \

  --variant $VCF_PATH/haplotypecaller_full.g.vcf.gz \

  --output $VCF_PATH/haplotypecaller_full.vcf.gz

# Apply basic GATK hard filters

echo "=== Applying GATK hard filters" >> $META_REPORT

$BCFTOOLS_PATH/bcftools view \

  --include 'QUAL>20 & INFO/QD>2 & INFO/MQ>40 & INFO/FS<60 & INFO/SOR<3

& INFO/MQRankSum>-12.5 & INFO/ReadPosRankSum>-8 & TYPE="snp"' \

  --output-type z \

  --output-file $VCF_PATH/haplotypecaller_filtered_snp.vcf.gz \

  $VCF_PATH/haplotypecaller_full.vcf.gz &

# The normalization step is potentially not required but it is harmless

$BCFTOOLS_PATH/bcftools view \

  --include 'QUAL>20 & INFO/QD>2 & INFO/ReadPosRankSum>-20 & INFO/InbreedingCoeff>-0.8 &

INFO/FS<200 & INFO/SOR<10 & TYPE∼"indel"' \

  $VCF_PATH/haplotypecaller_full.vcf.gz | \

  $BCFTOOLS_PATH/bcftools norm \

  --fasta-ref $BWA_INDEX \

  --output-type z \

  --output $VCF_PATH/haplotypecaller_filtered_norm_indel.vcf.gz &

wait

echo "=== Merging GATK filtered SNPs and INDELs" >> $META_REPORT

$GATK_PATH/gatk MergeVcfs \

  --INPUT $VCF_PATH/haplotypecaller_filtered_snp.vcf.gz \

  --INPUT $VCF_PATH/haplotypecaller_filtered_norm_indel.vcf.gz \

  --OUTPUT $VCF_PATH/haplotypecaller_filtered_norm.vcf.gz \

  --QUIET

rm $VCF_PATH/haplotypecaller_filtered_snp.vcf.gz \

 $VCF_PATH/haplotypecaller_filtered_norm_indel.vcf.gz

#echo "=== Finished!"

echo "=== Finished!" >> $META_REPORT

### Variant calling with FreeBayes

**Timing: 5 h**

This section presents the variant calling and filtering steps with FreeBayes. Its output is a VCF file with filtered (basic filters) variants called with FreeBayes.

In comparison with GATK HaplotypeCaller, the model behind FreeBayes does not require BQSR (therefore it is faster), requires all samples processed altogether and at once in the same command (using read groups and the RG tag to distinguish them) and does not operate directly on HaplotypeCaller genomic intervals. These have to be recalculated and reformatted to the BED format for FreeBayes parallelization.

Although there are many alternatives to run exome analysis with FreeBayes in an efficient way (e.g., parallelization of exome kit capture intervals or running each sample on the background or even using GNU parallel), we propose the following protocol (“intervals” are the capture kit genomic intervals recreated with GATK SplitIntervals for FreeBayes):

14. Rerun GATK SplitIntervals to create FreeBayes specific intervals for parallelization.
15. Create a list file with the individual interval filenames.
16. Create a list file with the individual BAM filenames.
17. For each interval, run FreeBayes jointly for all samples to create a VCF file for that interval.
18. Merge the produced multi-sample VCFs for each interval into one multi-sample VCF file using vcflib.
19. Using bcftools and R, determine upper quality (QUAL) and depth (DP) cutoffs based on the respective distributions (assuming initial QUAL>20).
20. Using bcftools apply the filters of (6).
21. Using vcflib decompose the complex variants.
22. Using bcftools normalize INDELs and produce the final VCF.
23. Cleanup the computation environment.

Summaries for all steps (including background processes) are recorded in a “report” file for general supervision. The following shell script template can be used to run the above protocol:

#!/bin/bash

export VCF_PATH=$HOME_PATH/vcf

BAM_PATH=$HOME_PATH/bam

CAPTURE_KIT=$RESOURCES_PATH/panel/Agilent_SureSelect_All_Exon_V2.bed

INTERVAL_LIST_PATH=$RESOURCES_PATH/resources/interval_scatter_bed

BWA_INDEX=$RESOURCES_PATH/hs37d5/hs37d5.fa

CORES=32

PADDING=50

META_REPORT=$HOME_PATH/reports/freebayes_current.log

echo "=== Splitting intervals" > $META_REPORT

if [ -d $INTERVAL_LIST_PATH ]

then

 echo " Cleaning previous intervals" >> $META_REPORT

 rm -r $INTERVAL_LIST_PATH

fi

mkdir -p $INTERVAL_LIST_PATH

# Firstly split exome intervals for parallel freebayes

$GATK_PATH/gatk SplitIntervals \

  --reference $BWA_INDEX \

  --intervals $CAPTURE_KIT \

  --interval-padding $PADDING \

  --scatter-count $CORES \

  --extension .pre \

  --output $INTERVAL_LIST_PATH \

  --QUIET

echo "=== Converting intervals" >> $META_REPORT

for INTERVAL in `ls $INTERVAL_LIST_PATH`

do

 BED=`basename $INTERVAL | sed s/\.pre//`

 INTERVAL_FILE=$INTERVAL_LIST_PATH/$INTERVAL

 grep -vP "ˆ@" $INTERVAL_FILE | awk '{print $1"\t"$2"\t"$3}' > \

  $INTERVAL_LIST_PATH/$BED".bed" &

done

# Wait and clear intermediate intervals

wait

rm $INTERVAL_LIST_PATH/∗.pre

# Prepare BAM file list for freebayes

echo "=== Preparing BAM file list" >> $META_REPORT

BAMLIST=/media/raid/tmp/tmp/medex/scripts/bamlist.txt

if [ -f $BAMLIST ]

then

 rm $BAMLIST

fi

for FILE in `ls $BAM_PATH/∗_fixmate\.bam`

do

 SAMPLE=`basename $FILE | sed s/_fixmate\.bam//`

 BAM=$BAM_PATH/$SAMPLE/$SAMPLE".bam"

 echo "$BAM" >> $BAMLIST

done

echo "=== Calling variants with FreeBayes" >> $META_REPORT

if [ -d $VCF_PATH/fb_parts ]

then

 rm -r $VCF_PATH/fb_parts

fi

mkdir -p $VCF_PATH/fb_parts

for TARGET in `ls $INTERVAL_LIST_PATH`

do

 NAME=`basename $TARGET | sed s/\.bed//`

 echo "Processing interval list $NAME" >> $META_REPORT

 INTERVAL=$INTERVAL_LIST_PATH/$TARGET

 $FREEBAYES_PATH/freebayes \

  --fasta-reference $BWA_INDEX \

  --bam-list $BAMLIST \

  --targets $INTERVAL \

  --vcf $VCF_PATH/fb_parts/$NAME".vcf" &

done

# Wait before gathering the results

wait

echo "=== Merging VCFs" >> $META_REPORT

cat $VCF_PATH/∗.vcf | \

 $VCFLIB_PATH/scripts/vcffirstheader | \

 $VCFLIB_PATH/bin/vcfstreamsort -w 1000 | \

 $VCFLIB_PATH/bin/vcfuniq > \

 $VCF_PATH/all_samples_freebayes.vcf

echo "=== Compressing and indexing final VCF" >> $META_REPORT

$HTSLIB_PATH/bgzip $VCF_PATH/freebayes_full.vcf

$HTSLIB_PATH/tabix $VCF_PATH/freebayes_full.vcf.gz

### Basic filtering before decomposing and normalization

# Determine a quality and depth cutoff pre-filter based on 99th percentile of

# the respective distributions

echo "=== Determining QUAL and DP hard pre-filters" >> $META_REPORT

$BCFTOOLS_PATH/bcftools query \

  --include 'QUAL>20' \

  --format '%QUAL\n' $VCF_PATH/freebayes_full.vcf.gz > quals.tmp &

$BCFTOOLS_PATH/bcftools query \

  --include 'QUAL>20' \

  --format '%INFO/DP\n' $VCF_PATH/freebayes_full.vcf.gz | \

  awk -F "," '{print $1}' > $VCF_PATH/dps.tmp &

wait

Rscript -e '

  vp <- Sys.getenv("VCF_PATH")

  dps <- as.numeric(readLines(file.path(vp,"dps.tmp")));

  quals <- as.numeric(readLines(file.path(vp,"quals.tmp")));

  qudp <- unname(round(quantile(dps,0.99)));

  ququ <- unname(quantile(quals,0.99));

  write(qudp,file.path(vp,"dpt.tmp"));

  write(ququ,file.path(vp,"qut.tmp"));

'

QUALUP=`cat $VCF_PATH/qut.tmp`

DPUP=`cat $VCF_PATH/dpt.tmp`

rm $VCF_PATH/qut.tmp $VCF_PATH/dpt.tmp $VCF_PATH/dps.tmp $VCF_PATH/quals.tmp

# Apply the filters, decompose complex variants and normalize

echo "=== Applying filters and normalizing" >> $META_REPORT

$BCFTOOLS_PATH/bcftools view \

  --include 'QUAL>20 & INFO/DP>10 & QUAL<'$QUALUP' & INFO/DP<'$DPUP' &

(QUAL/(INFO/DP))>2' $VCF_PATH/freebayes_full.vcf.gz | \

  $VCFLIB_PATH/bin/vcfallelicprimitives -kg | \

  $BCFTOOLS_PATH/bcftools norm \

  --fasta-ref $BWA_INDEX \

  --output-type z \

  --output $VCF_PATH/freebayes_filtered_norm.vcf.gz

$HTSLIB_PATH/tabix $VCF_PATH/freebayes_filtered_norm.vcf.gz

echo "=== Finished!" >> $META_REPORT

### Variant calling with DeepVariant

**Timing: 1 h**

The model behind DeepVariant is similar to FreeBayes regarding BQSR, therefore not needing it. DeepVariant requires the RG tag (read groups) and splits the capture kit in BED format. The splitting is done internally, so no manual split required from the user for parallelization based on the capture kit genomic intervals. The output of this part is a VCF file with filtered variants called with DeepVariant. Based on DeepVariant authors, we propose the following protocol:

24. For each sample run DeepVariant and create a gVCF and a VCF file.
25. Create a list file with gVCF outputs of DeepVariant for input to DNA Nexus GLnexus.
26. Run GLnexus on the DeepVariant gVCFs to consolidate the gVCFs into one final population VCF file.
27. Using bcftools filter the variants with QUAL<20 and normalize.

The DeepVariant pipeline is pretty well-defined and quite automated, leaving few steps for the user which essentially come down to variant filtering (which again is not complex). Summaries for all steps (including background processes) are recorded in a “report” file for general supervision. The following shell script template can be used to run the above protocol:

#!/bin/bash

export VCF_PATH=$HOME_PATH/vcf

BAM_PATH=$HOME_PATH/bam

CAPTURE_KIT_DIR=$RESOURCES_PATH/resources/panel

CAPTURE_KIT=$RESOURCES_PATH/panel/Agilent_SureSelect_All_Exon_V2.bed

DV_VERSION=0.9.0

BWA_INDEX_DIR=$RESOURCES_PATH/hs37d5

BWA_INDEX=$RESOURCES_PATH/hs37d5/hs37d5.fa

CORES=32

META_REPORT=$HOME_PATH/reports/deepvariant_current.log

echo "=== Calling variants" > $META_REPORT

for FILE in `ls $BAM_PATH/∗_fixmate.bam`

do

 SAMPLE=`basename $FILE | sed s/_fixmate\.bam//`

 echo "Processing $SAMPLE" >> $META_REPORT

 BAM=$BAM_PATH/$SAMPLE".bam"

 docker run \

 -v "$BAM_PATH":"/data" \

 -v "$BWA_INDEX_DIR":"/reference" \

 -v "$CAPTURE_KIT_DIR":"/capture_kit" \

 google/deepvariant:$DV_VERSION \

 /opt/deepvariant/bin/run_deepvariant \

 --model_type=WES \

 --ref="/reference/hs37d5.fa" \

 --reads="/data/$SAMPLE.bam" \

 --regions="/capture_kit/Agilent_SureSelect_All_Exon_V2.bed" \

 --output_vcf="/data/$SAMPLE/$SAMPLE'_DV.vcf'" \

 --output_gvcf="/data/$SAMPLE/$SAMPLE'_DV.g.vcf'" \

 --num_shards=$CORES

done

echo "=== Creating list of gVCF files" >> $META_REPORT

for FILE in `ls $BAM_PATH/∗_fixmate.bam`

do

 SAMPLE=`basename $FILE | sed s/_fixmate\.bam//`

 GVCF=`readlink -f $BAM_PATH/$SAMPLE/$SAMPLE"_DV.g.vcf"`

 echo "$GVCF" >> $VCF_PATH/deepvariant_gvcf_list.txt

done

echo "=== Gathering gVCFs" >> $META_REPORT

rm -r GLnexus.DB

$GLNEXUS_PATH/glnexus_cli \

  --config DeepVariantWES \

  --bed $CAPTURE_KIT \

  --list $VCF_PATH/deepvariant_gvcf_list.txt \

  --threads $CORES | \

  $BCFTOOLS_PATH/bcftools view --include 'QUAL>=20' - | \

  $BCFTOOLS_PATH/bcftools norm \

  --fasta-ref $BWA_INDEX \

  --output-type z \

  --output $VCF_PATH/deepvariant_filtered_norm.vcf.gz

$HTSLIB_PATH/tabix $VCF_PATH/deepvariant_filtered_norm.vcf.gz

echo "=== Finished!" >> $META_REPORT

### Variant annotation

**Timing: 9 h 30 min**

In this section the output of each variant caller is annotated with additional elements such as variant impacts and frequencies of known variants in major population studies. The output of this part is one annotated VCF file for each variant caller.

28. Using SnpEff and SnpSift, annotate the findings with basic information including:
  a. Genomic location (gene, exon, etc.).
  b. Impact prediction based on the Sequence Ontology and the Sequence Variant Nomenclature.
  c. Known variant IDs from dbSNP.
  d. Various pathogenicity prediction scores and other SNP metrics from dbNSFP.
  e. Population study variant frequencies from gnomAD.

**CRITICAL:** It is assumed that the required resources for SnpEff and SnpSift are in place (see also the “before you begin” section). Prior to using SnpEff and SnpSift, a SnpEff database for our genome of interest must be downloaded (see script below).

The following shell script template can be used for annotation of the final (filtered) outputs from each variant caller:

#!/bin/bash

export VCF_PATH=$HOME_PATH/vcf

DBSNP_FILE=$RESOURCES_PATH/dbSNP/dbSNP151.vcf.gz

DBNSFP_FILE=$RESOURCES_PATH/dbNSFP/dbNSFP2.9.3.txt.gz

GNOMAD_FILE=$RESOURCES_PATH/gnomAD/gnomad.exomes.r2.1.1.sites.vcf.bgz

if [ ! -d $SNPEFF_PATH/data ]

then

 java -jar $SNPEFF_PATH/snpEff.jar download GRCh37.75

fi

## Haplotype Caller

# Variant effect annotation

java -Xmx4096m -jar $SNPEFF_PATH/snpEff.jar ann \

 -v -noLog -noStats -noLof GRCh37.75 \

 $VCF_PATH/haplotypecaller_filtered_norm.vcf.gz >

$VCF_PATH/haplotypecaller_filtered_norm_eff.vcf

$HTSLIB_PATH/bgzip $VCF_PATH/haplotypecaller_filtered_norm_eff.vcf

$HTSLIB_PATH/tabix $VCF_PATH/haplotypecaller_filtered_norm_eff.vcf.gz

# Annotation with dbSNP

java -Xmx4096m -jar $SNPEFF_PATH/SnpSift.jar annotate \

 -v -id $DBSNP_FILE \

 $VCF_PATH/haplotypecaller_filtered_norm_eff.vcf.gz >

$VCF_PATH/haplotypecaller_filtered_norm_eff_dbsnp.vcf

$HTSLIB_PATH/bgzip

 $VCF_PATH/haplotypecaller_filtered_norm_eff_dbsnp.vcf.gz

$HTSLIB_PATH/tabix $VCF_PATH/haplotypecaller_filtered_norm_eff_dbsnp.vcf.gz

# Annotation with dbNSFP

java -Xmx4096m -jar $SNPEFF_PATH/SnpSift.jar dbnsfp \

 -v -m -db $DBNSFP_FILE \

 $VCF_PATH/haplotypecaller_filtered_norm_eff_dbsnp.vcf.gz >

$VCF_PATH/haplotypecaller_filtered_norm_eff_dbsnp_dbnsfp.vcf

 $HTSLIB_PATH/bgzip $VCF_PATH/haplotypecaller_filtered_norm_eff_dbsnp_dbnsfp.vcf

$HTSLIB_PATH/tabix

$VCF_PATH/haplotypecaller_filtered_norm_eff_dbsnp_dbnsfp.vcf.gz

# Annotation with gnomAD

java -Xmx4096m -jar $SNPEFF_PATH/SnpSift.jar annotate \

 -v $GNOMAD_FILE \

 $VCF_PATH/haplotypecaller_filtered_norm_eff_dbsnp_dbnsfp.vcf.gz >

$VCF_PATH/haplotypecaller_filtered_norm_eff_dbsnp_dbnsfp_gnomad.vcf

 $HTSLIB_PATH/bgzip

$VCF_PATH/haplotypecaller_filtered_norm_eff_dbsnp_dbnsfp_gnomad.vcf

$HTSLIB_PATH/tabix $VCF_PATH/haplotypecaller_filtered_norm_eff_dbsnp_dbnsfp_gnomad.vcf.gz

# FreeBayes

# Variant effect annotation

java -Xmx4096m -jar $SNPEFF_PATH/snpEff.jar ann \

 -v -noLog -noStats -noLof GRCh37.75 \

 $VCF_PATH/freebayes_filtered_norm.vcf.gz > $VCF_PATH/freebayes_filtered_norm_eff.vcf

 $HTSLIB_PATH/bgzip $VCF_PATH/freebayes_filtered_norm_eff.vcf

 $HTSLIB_PATH/tabix \

 $VCF_PATH/freebayes_filtered_norm_eff.vcf.gz

# Annotation with dbSNP

java -Xmx4096m -jar $SNPEFF_PATH/SnpSift.jar annotate \

 -v -id $DBSNP_FILE \

 $VCF_PATH/freebayes_filtered_norm_eff.vcf.gz > $VCF_PATH/freebayes_filtered_norm_eff_dbsnp.vcf

 $HTSLIB_PATH/bgzip\

 $VCF_PATH/freebayes_filtered_norm_eff_dbsnp.vcf.gz

 $HTSLIB_PATH/tabix \

 $VCF_PATH/freebayes_filtered_norm_eff_dbsnp.vcf.gz

# Annotation with dbNSFP

java -Xmx4096m -jar $SNPEFF_PATH/SnpSift.jar dbnsfp \

 -v -m -db $DBNSFP_FILE \

 $VCF_PATH/freebayes_filtered_norm_eff_dbsnp.vcf.gz >

$VCF_PATH/freebayes_filtered_norm_eff_dbsnp_dbnsfp.vcf

 $HTSLIB_PATH/bgzip\

 $VCF_PATH/freebayes_filtered_norm_eff_dbsnp_dbnsfp.vcf \

 $HTSLIB_PATH/tabix \

 $VCF_PATH/freebayes_filtered_norm_eff_dbsnp_dbnsfp.vcf.gz

# Annotation with gnomAD

java -Xmx4096m -jar $SNPEFF_PATH/SnpSift.jar annotate \

 -v $GNOMAD_FILE \

 $VCF_PATH/freebayes_filtered_norm_eff_dbsnp_dbnsfp.vcf.gz >

$VCF_PATH/freebayes_filtered_norm_eff_dbsnp_dbnsfp_gnomad.vcf

 $HTSLIB_PATH/bgzip\

 $VCF_PATH/freebayes_filtered_norm_eff_dbsnp_dbnsfp_gnomad.vcf

 $HTSLIB_PATH/tabix \

 $VCF_PATH/freebayes_filtered_norm_eff_dbsnp_dbnsfp_gnomad.vcf.gz

## Deep Variant

# Variant effect annotation

java -Xmx4096m -jar $SNPEFF_PATH/snpEff.jar ann \

 -v -noLog -noStats -noLof GRCh37.75 \

 $VCF_PATH/deepvariant_filtered_norm.vcf.gz >

$VCF_PATH/deepvariant_filtered_norm_eff.vcf.gz

 $HTSLIB_PATH/bgzip \

 $VCF_PATH/deepvariant_filtered_norm_eff.vcf

 $HTSLIB_PATH/tabix \

 $VCF_PATH/deepvariant_filtered_norm_eff.vcf.gz

# Annotation with dbSNP

java -Xmx4096m -jar $SNPEFF_PATH/SnpSift.jar annotate \

 -v -id $DBSNP_FILE \

 $VCF_PATH/deepvariant_filtered_norm_eff.vcf.gz >

$VCF_PATH/deepvariant_filtered_norm_eff_dbsnp.vcf

 $HTSLIB_PATH/bgzip\

 $VCF_PATH/deepvariant_filtered_norm_eff_dbsnp.vcf

 $HTSLIB_PATH/tabix \

 $VCF_PATH/deepvariant_filtered_norm_eff_dbsnp.vcf.gz

# Annotation with dbNSFP

java -Xmx4096m -jar $SNPEFF_PATH/SnpSift.jar dbnsfp \

 -v -m -db $DBNSFP_FILE \

 $VCF_PATH/deepvariant_filtered_norm_eff_dbsnp.vcf.gz >

$VCF_PATH/deepvariant_filtered_norm_eff_dbsnp_dbnsfp.vcf

 $HTSLIB_PATH/bgzip\

 $VCF_PATH/deepvariant_filtered_norm_eff_dbsnp_dbnsfp.vcf.gz

 $HTSLIB_PATH/tabix \

 $VCF_PATH/deepvariant_filtered_norm_eff_dbsnp_dbnsfp.vcf.gz

# Annotation with gnomAD

java -Xmx4096m -jar $SNPEFF_PATH/SnpSift.jar annotate \

 -v $GNOMAD_FILE \

 $VCF_PATH/deepvariant_filtered_norm_eff_dbsnp_dbnsfp.vcf.gz >

$VCF_PATH/deepvariant_filtered_norm_eff_dbsnp_dbnsfp_gnomad.vcf.gz

 $HTSLIB_PATH/bgzip\

 $VCF_PATH/deepvariant_filtered_norm_eff_dbsnp_dbnsfp_gnomad.vcf

 $HTSLIB_PATH/tabix \

 $VCF_PATH/deepvariant_filtered_norm_eff_dbsnp_dbnsfp_gnomad.vcf.gz

# Remove intermediate files

rm $VCF_PATH/haplotypecaller_filtered_norm_eff.vcf.gz \

 $VCF_PATH/haplotypecaller_filtered_norm_eff.vcf.gz.tbi \

 $VCF_PATH/haplotypecaller_filtered_norm_eff_dbsnp.vcf.gz \

 $VCF_PATH/haplotypecaller_filtered_norm_eff_dbsnp.vcf.gz.tbi \

 $VCF_PATH/freebayes_filtered_norm_eff.vcf.gz \

 $VCF_PATH/freebayes_filtered_norm_eff.vcf.gz.tbi \

 $VCF_PATH/freebayes_filtered_norm_eff_dbsnp.vcf.gz \

 $VCF_PATH/freebayes_filtered_norm_eff_dbsnp.vcf.gz.tbi \

 $VCF_PATH/deepvariant_filtered_norm_eff.vcf.gz \

 $VCF_PATH/deepvariant_filtered_norm_eff.vcf.gz.tbi \

 $VCF_PATH/deepvariant_filtered_norm_eff_dbsnp.vcf.gz \

 $VCF_PATH/deepvariant_filtered_norm_eff_dbsnp.vcf.gz.tbi

### Variant callset consolidation

**Timing: 20 min**

In this section, we consolidate the variant calls from the three different callers. The output consists of several VCF files with unique and combined annotated variants for each caller as well as common variants between all callers and between pairs of callers. The output also contains the genotypes returned by each caller.

29. Consolidate variant calls using bcftools.

#!/bin/bash

export VCF_PATH=$HOME_PATH/vcf

# 1

$BCFTOOLS_PATH/bcftools isec \

  --prefix 1 \

  --output-type z \

  --nfiles ∼100 \

  --collapse none \

  $VCF_PATH/haplotypecaller_filtered_norm_eff_dbsnp_dbnsfp.vcf.gz \

  $VCF_PATH/freebayes_filtered_norm_eff_dbsnp_dbnsfp.vcf.gz \

  $VCF_PATH/deepvariant_filtered_norm_eff_dbsnp_dbnsfp.vcf.gz

AREA1=`cat ./1/sites.txt | wc -l`

echo $AREA1

# 2

$BCFTOOLS_PATH/bcftools isec \

  --prefix 2 \

  --output-type z \

  --nfiles ∼010 \

  --collapse none \

  $VCF_PATH/haplotypecaller_filtered_norm_eff_dbsnp_dbnsfp.vcf.gz \

  $VCF_PATH/freebayes_filtered_norm_eff_dbsnp_dbnsfp.vcf.gz \

  $VCF_PATH/deepvariant_filtered_norm_eff_dbsnp_dbnsfp.vcf.gz

AREA2=`cat ./2/sites.txt | wc -l`

echo $AREA2

# 3

$BCFTOOLS_PATH/bcftools isec \

  --prefix 3 \

  --output-type z \

  --nfiles ∼001 \

  --collapse none \

  $VCF_PATH/haplotypecaller_filtered_norm_eff_dbsnp_dbnsfp.vcf.gz \

  $VCF_PATH/freebayes_filtered_norm_eff_dbsnp_dbnsfp.vcf.gz \

  $VCF_PATH/deepvariant_filtered_norm_eff_dbsnp_dbnsfp.vcf.gz

AREA3=`cat ./3/sites.txt | wc -l`

echo $AREA3

# 4

$BCFTOOLS_PATH/bcftools isec \

  --prefix 4 \

  --output-type z \

  --nfiles ∼110 \

  --collapse none \

  $VCF_PATH/haplotypecaller_filtered_norm_eff_dbsnp_dbnsfp.vcf.gz \

  $VCF_PATH/freebayes_filtered_norm_eff_dbsnp_dbnsfp.vcf.gz \

  $VCF_PATH/deepvariant_filtered_norm_eff_dbsnp_dbnsfp.vcf.gz

AREA4=`cat ./4/sites.txt | wc -l`

echo $AREA4

# 5

$BCFTOOLS_PATH/bcftools isec \

  --prefix 5 \

  --output-type z \

  --nfiles ∼011 \

  --collapse none \

  $VCF_PATH/haplotypecaller_filtered_norm_eff_dbsnp_dbnsfp.vcf.gz \

  $VCF_PATH/freebayes_filtered_norm_eff_dbsnp_dbnsfp.vcf.gz \

  $VCF_PATH/deepvariant_filtered_norm_eff_dbsnp_dbnsfp.vcf.gz

AREA5=`cat ./5/sites.txt | wc -l`

echo $AREA5

# 6

$BCFTOOLS_PATH/bcftools isec \

  --prefix 6 \

  --output-type z \

  --nfiles ∼101 \

  --collapse none \

  $VCF_PATH/haplotypecaller_filtered_norm_eff_dbsnp_dbnsfp.vcf.gz \

  $VCF_PATH/freebayes_filtered_norm_eff_dbsnp_dbnsfp.vcf.gz \

  $VCF_PATH/deepvariant_filtered_norm_eff_dbsnp_dbnsfp.vcf.gz

AREA6=`cat ./6/sites.txt | wc -l`

echo $AREA6

# 7

$BCFTOOLS_PATH/bcftools isec \

  --prefix 7 \

  --output-type z \

  --nfiles ∼111 \

  --collapse none \

  $VCF_PATH/haplotypecaller_filtered_norm_eff_dbsnp_dbnsfp.vcf.gz \

  $VCF_PATH/freebayes_filtered_norm_eff_dbsnp_dbnsfp.vcf.gz \

  $VCF_PATH/deepvariant_filtered_norm_eff_dbsnp_dbnsfp.vcf.gz

AREA7=`cat ./7/sites.txt | wc -l`

echo $AREA7

***Note:*** A challenging issue when using variant callers is how to summarize and consolidate different DNA variant callsets from different callers into one summarized result. Major challenges for consolidation include the decision on which of the reported variant call metrics reported in VCF file(s) from each caller will be included in the final callset (e.g., which QUAL, which DP etc.) and the level at which the variants from different callers should be considered identical or nearly identical. Regarding the latter, two common questions are should they be considered identical if they share the same genomic coordinates or start position, or, should they be considered identical if they share both positions and alleles?

Fortunately, bcftools offer functions to experiment with the many options that exist to consolidate the callsets. We have chosen to intersect the callsets and consider the overlapping variants identical if they share both genomic position and alleles. We perform the various intersections using bcftools and for each intersection we perform three operations in order to retain all the metrics for each caller but on the intersected (shared) variants. From the produced callsets, the most interesting one to begin the exploration should be the #7 which corresponds to the common variants between all the three callers we have used.

### Visualization and further post-processing

**Timing: 1 h**

As with most high-throughput techniques, the final processed data cannot be fully denoised, and some false positives and artifacts are always to be expected. One popular way of further assessing the quality of the produced data is visualization. In this section we describe how the variant callsets can be visualized in two ways. Firstly, by simultaneous loading and visualization of the results (VCF files) and raw data (BAM files) in a genome browser such as IGV and secondly, with a Venn diagram to qualitatively visualize overlaps between callsets. In addition, we briefly discuss the need for additional filtering steps according to the application of the WES experiment, for example clinical settings or population studies.

This protocol step comprises two substeps:

30. Generation of a Venn diagram to depict common and unique variants across the three callsets.

**Figure 1:**
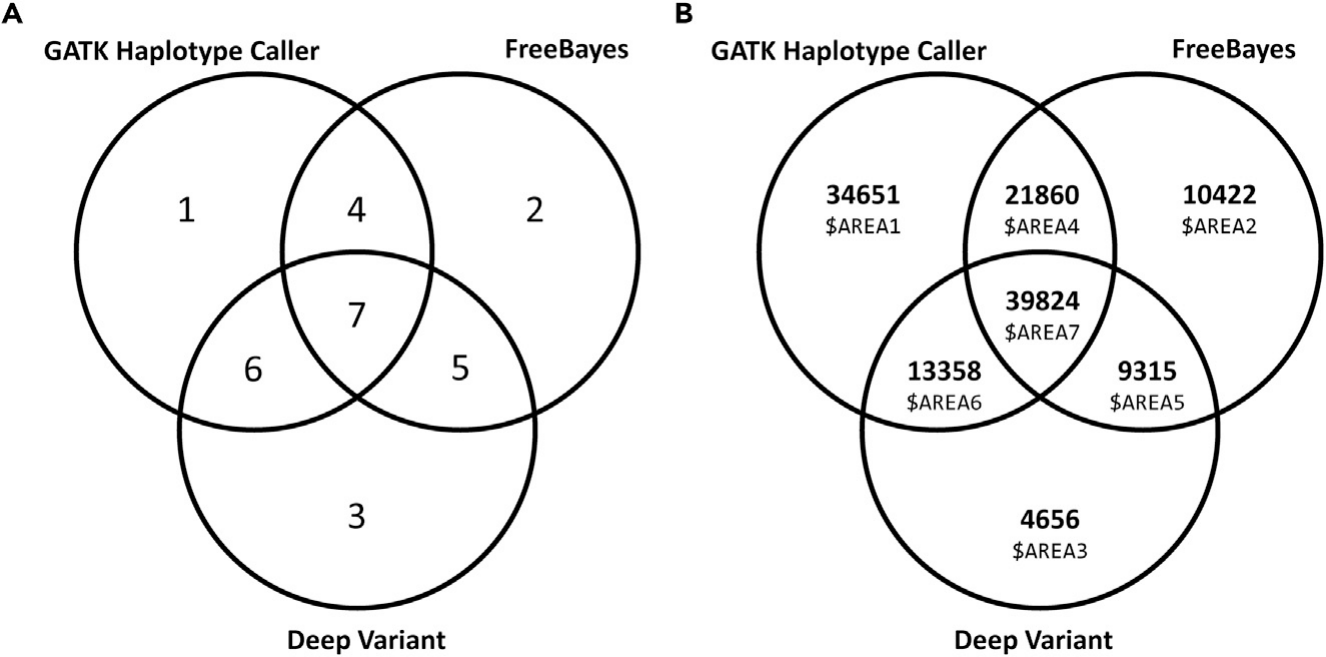
Venn diagram visualization of the three callsets (A) Numbering of the Venn overlapping and unique areas which correspond to specific callsets created with bcftools. (B) The same Venn diagram where the areas depicted in (A) have been filled with the actual number of variants resulting from the application of the protocol to the data presented in the article, accompanied by the shell variable names presented in the code in part “variant callset consolidation” and step 29.

A 3-way Venn diagram contains seven areas (Figure 1A). Each area is numbered according to the number in the comment section directly above each $BCFTOOLS_PATH/bcftools isecin the commands presented in step 7. For example, the number of variants in area 1, is given by the $BCFTOOLS_PATH/bcftools isec command below the line containing #1. By using the outcome of echoing variable X in the same command-line set, the user can fill the numbers required for the completion of the Venn diagram (Figure 1B).

31. Visualization of the callsets in the IGV genome browser.
  At the end of parts “preparation of BAM files”, “signal visualization”, “variant calling with GATK HaplotypeCaller”, “variant calling with FreeBayes”, “variant calling with DeepVariant” the following files were produced respectively:
  a. At the end of part “preparation of BAM files”, read alignment files in BAM format.
  b. At the end of step “signal visualization”, WES signal visualization files in BigWig format.
  c. At the end of step “variant calling with GATK HaplotypeCaller”, a filtered VCF file with GATK Haplotype Caller results.
  d. At the end of step “variant calling with FreeBayes”, a filtered VCF file with FreeBayes results.
  e. At the end of step “variant calling with DeepVariant”, a filtered VCF file with DeepVariant results.

These files can be loaded in the IGV genome browser for visualization of the results with the following steps.

32. Download and install the IGV genome browser.
33. Open the IGV genome browser.
34. From the *Genomes* menu select *Load Genome From Server.*
35. Select *Human (1 kg, b37 + decoy)* or *Human (b37)* and click OK.
36. From the File menu select *Load from File.*
37. Select one or more from the BAM files created at the end of the part “preparation of BAM files”.
38. From the File menu select *Load from File.*
39. Select one or more from the BigWig files created at the end of the part “signal visualization”.
40. From the File menu select *Load from File.*
41. Select the three variant callsets generated at the end of the parts “variant calling with GATK HaplotypeCaller”, “variant calling with FreeBayes” and “variant calling with DeepVariant”.

Following the steps (32)–(41), the user should be able to visualize variant callsets and supporting information such as overall signal and reads supporting each variant call. The user can navigate through the callsets using the respective IGV controls (zoom in and out, navigate to specific areas by chromosomal coordinates etc.).

## Expected outcomes

WES comprises a well-defined and much promising NGS technique which has been successfully deployed during the past few years with many applications in research and the clinic. The output of WES is typically a (long) list of DNA variations detected given an input DNA sample such as from a patient, when compared to a reference genome. As with every major high-throughput technique, the output is prone to noise and potential errors which require special handling in order to be filtered out and reduce false positives. The proposed protocol may aid achieve this through careful data filtering and preparation followed by variant calling with three established variant callers and subsequent (clinical) annotation and consolidation of the results.

Regarding the actual potential reduction in false positives, an estimation can be provided based on recent studies where multiple variant callers are evaluated (Barbitoff et al., 2022; Lin et al., 2018) and combined (Zhao et al., 2020). In (Zhao et al., 2020), the authors benchmarked GATK, the Illumina DRAGEN-based caller and DeepVariant using human genome data and it was shown that the average F1-score for SNP detection across 4 datasets is 0.990 for GATK without Variant Quality Score Recalibration and 0.969 for GATK with Variant Quality Score Recalibration. Furthermore, in the same study it was shown that the combination of GATK with DeepVariant leads to higher F1-scores. The average F1-score for the combined methodology returned was 0.993 on average, suggesting that the combination of methods is expected to lead to more accurate SNP calling results. In addition, in (Lin et al., 2018), the comparison of GATK with DeepVariant, when applied on the analysis of trios, showed that DeepVariant made fewer calls, but with a lower false positive rate. In addition, in (Barbitoff et al., 2022), the F1-scores calculated for the three methods when applied on Whole Exome Sequencing were 0.996 for DeepVariant, 0.985 for GATK and 0.987 for FreeBayes. Based on these results and the aforementioned results regarding algorithm combination, we expect the overall F1-score to be >0.996. Last but not least, in our experience GATK tends to return more variants than the other two methodologies (Figure 1B) even after the application of the best-practice filters, suggesting a potential higher rate of false discoveries.

The proposed protocol produces outputs at various processing steps and at various levels. Specifically, the main outputs are quality controlled raw data in FASTQ format, alignments to the reference genome in BAM format, variant callsets from each caller in VCF format and annotated and consolidated variant callsets in VCF format.

## Limitations

Despite the detailed description of the protocol steps, installation of prerequisite software and script templates that can be almost used out of the box by the user, there are cases where the input of a computer expert or a trained bioinformatician may be needed. Such cases could include the installation of tools requiring system-level access such as Docker, or the navigation among GATK available tools and commands. In addition, the described protocol assumes a Linux-based system and some basic skills in using the command line. Although the latter skills are not extensive and the protocol steps are very detailed, some users may find it difficult to follow. Another limitation, and also the reason for which command-line skills are required, is the fact that most of the required tools are well-behaved mostly in Linux environments. Executing them on other operating systems (such as Windows) is not prohibitive but require substantial skills and software prerequisites as most of them would require to be re-compiled from source code. On the other hand, most of them are available out of the box for Linux environments. Additional limitations may have to do with available computational power and storage resources. While most tools are flexible and running them with a few or even one core is possible -albeit much slower- the required annotation resources require available storage. However, most laboratories engaged in WES should have storage resources available. Finally, although the usage of multiple variant callers depending on different underlying statistical models may reduce related introduced biases and thus reduce false positives, visualization of the end-result is also required to derive final conclusions especially in clinical settings. Such visualization is possible through dedicated genome browsers such as the IGV, which operate on local systems and can load simultaneously WES signal (BigWig files), read alignment files (BAM files) and the called variants (VCF files). In this way, the analyst can verify – for some representative cases at least – the validity of the presence of a variant in all three callsets and if there are false calls based on aligned reads support. All this information is available within IGV.

## Troubleshooting

### Problem 1

The hardware I have at my disposal to run the protocol is not adequate to guarantee performance.

### Potential solution

Generally, the vast majority of the tools used in the protocol can run in single core, lower-end systems such as a medium to high-end laptop. The user should try and drastically reduce the number of cores to use (denoted by the “CORES” environmental variable where applicable) and also reduce the number of compute jobs executed in the background, that is remove ampersands (& symbol) at the end of certain commands throughout the template scripts. The protocol will be completed but the timings will increase at rates 50%–1000%.

### Problem 2

The protocol describes the variant calling procedure with paired-end sequencing data. I want to execute the protocol with single-end sequencing data.

### Potential solution

The only steps slightly changing are the ones regarding basic quality control, alignment to the reference genome and the BAM file preprocessing which becomes shorter. We provide additional template scripts for this process in the GitHub repository accompanying this article.

### Problem 3

I cannot find the coordinate files for the exome capture kit or I am not sure about the kit used in my experiment.

### Potential solution

In this unlikely event, the user may use a list of all the reference genome exons from a public resource such as RefSeq or Ensembl. Some biases are expected. The user must make sure that the downloaded exon coordinates correspond to the same reference genome version used in the alignment process (e.g., hg19).

### Problem 4

Variant calling with DeepVariant crashes.

### Potential solution

Try to change the following lines in bold.

docker run \

 -v "$BAM_PATH":"/data" \

 -v "$BWA_INDEX_DIR":"/reference" \

 -v "$CAPTURE_KIT_DIR":"/capture_kit" \

 google/deepvariant:$DV_VERSION \

 /opt/deepvariant/bin/run_deepvariant \

 --model_type=WES \

 --ref="/reference/hs37d5.fa" \

 --reads="/data/$SAMPLE.bam" \

 --regions="/capture_kit/Agilent_SureSelect_All_Exon_V2.bed" \

 **--output_vcf="/data/$SAMPLE/$SAMPLE'_DV.vcf'" \**

 **--output_gvcf="/data/$SAMPLE/$SAMPLE'_DV.g.vcf'" \**

 --num_shards=$CORES

with

docker run \

 -v "$BAM_PATH":"/data" \

 -v "$BWA_INDEX_DIR":"/reference" \

 -v "$CAPTURE_KIT_DIR":"/capture_kit" \

 google/deepvariant:$DV_VERSION \

 /opt/deepvariant/bin/run_deepvariant \

 --model_type=WES \

 --ref="/reference/hs37d5.fa" \

 --reads="/data/$SAMPLE.bam" \

 --regions="/capture_kit/Agilent_SureSelect_All_Exon_V2.bed" \

 **--output_vcf=/data/$SAMPLE/$SAMPLE'_DV.vcf' \**

 **--output_gvcf=/data/$SAMPLE/$SAMPLE'_DV.g.vcf' \**

 --num_shards=$CORES

### Problem 5

Some tools require administrative access, or as a user I have limitations in installing tools, or I have not enough allocated space.

### Potential solution

All of the tools and commands in this protocol do not assume administrative access, except from the installation of Docker, which however is bundled with most modern Linux systems. In the unlikely event of limited user access, advice from a system administrator should be sought. The same applied to additional space requirements.

### Problem 6

No variants are left after the filters applied to the FreeBayes result.

### Potential solution

It is possible according to the particularities of each dataset that such a case may arrive. In this unlikely event, the filtering thresholds should be lowered. The user should change the following lines from the code in step 7.

Rscript -e '

 vp <- Sys.getenv("VCF_PATH")

 dps <- as.numeric(readLines(file.path(vp,"dps.tmp")));

 quals <- as.numeric(readLines(file.path(vp,"quals.tmp")));

 qudp <- unname(round(quantile(dps,0.99)));

 ququ <- unname(quantile(quals,0.99));

 write(qudp,file.path(vp,"dpt.tmp"));

 write(ququ,file.path(vp,"qut.tmp"));

'

with the following:

Rscript -e '

 vp <- Sys.getenv("VCF_PATH")

 dps <- as.numeric(readLines(file.path(vp,"dps.tmp")));

 quals <- as.numeric(readLines(file.path(vp,"quals.tmp")));

 qudp <- unname(round(quantile(dps,0.90)));

 ququ <- unname(quantile(quals,0.90));

 write(qudp,file.path(vp,"dpt.tmp"));

 write(ququ,file.path(vp,"qut.tmp"));

'

It is possible that the user may have to experiment with the quantile values, for example even set from 0.90 to 0.75.

## Resource availability

### Lead contact

Further information and requests for resources and reagents should be directed to and will be fulfilled by the lead contact, Panagiotis Moulos (moulos@fleming.gr).

### Materials availability

This study did not generate new unique reagents.

## Data Availability

This protocol did not generate any new datasets. The sample data analyzed in this protocol can be found at SRA and using the links in the data retrieval box in the respective section as well as the key resources table. The code templates outlined through the article are available at https://github.com/moulos-lab/star_protocols_wes3x (https://doi.org/10.5281/zenodo.6491376).
